# Pathways linking health literacy to self-care in diabetic patients with physical disabilities: A moderated mediation model

**DOI:** 10.1371/journal.pone.0299971

**Published:** 2024-03-14

**Authors:** Hye Jin Nam, Ju Young Yoon

**Affiliations:** 1 College of Nursing, Seoul National University, Seoul, South Korea; 2 Research Institute of Nursing Science, Seoul National University, Seoul, South Korea; 3 Center for Human-Caring Nurse Leaders for the Future by Brain Korea 21 (BK 21) Four Project, College of Nursing, Seoul National University, Seoul, South Korea; Chinese Academy of Medical Sciences and Peking Union Medical College, CHINA

## Abstract

**Introduction:**

Health literacy is widely considered to be a determinant of self-care behavior in people with diabetes. However, the mechanisms underlying how health literacy is linked to self-care behaviors have not been clearly elucidated. The aim of the present study was to explore the mediating roles of access to healthcare, provider-patient interaction, motivation, self-efficacy in the effect of health literacy on diabetes self-care behaviors among diabetic patients with physical disabilities and investigate the moderating effect of age in a moderated mediation model.

**Methods:**

The online survey was participated by a total of 214 diabetic patients with physical disabilities from November to December 2021. The moderated mediation analysis was examined using the Hayes’ PROCESS macro modeling tool based on the bias-corrected bootstrapping method.

**Results:**

After controlling for education, the results yielded a significant indirect effect of health literacy on diabetes self-care through motivation and self-efficacy. A partially mediating relationship also was confirmed, as there is a positive direct effect of health literacy on diabetes self-care. Furthermore, age groups (i.e., age <40 and ≥ 40) functioned as a moderator of the mediating effects of motivation and self-efficacy between health literacy and diabetes self-care.

**Conclusion:**

This study emphasized the important role of motivation and self-efficacy which play in linking health literacy and self-care behavior, especially for younger diabetic patients with physical disabilities. In the light of these findings, a health-literacy tailored motivation and self-efficacy enhancing program may be key targets for interventions promoting diabetes self-care behaviors in people with physical disabilities.

## Introduction

An estimated 1 billion people live with disabilities worldwide, and health disparities for this population have increasingly been recognized [1]. Studies have shown that individuals with disabilities are more likely to report less access to adequate health care than people without disabilities, thereby engaging in unhealthy behaviors, such as smoking cigarettes and physical inactivity, with poorer overall health outcomes [2]. Studies on health determinants in this underserved population have largely been neglected despite having the higher health risk of people with disabilities.

Diabetes mellitus is one of the most prevalent chronic diseases in the disability population. Individuals living with disabilities have been determined to have a two times higher risk of diabetes than those without disabilities [3]. People with poorly controlled diabetes are at risk of serious complications, such as cardiovascular disease, nephropathy, retinopathy, and neuropathy, which results in premature death. The most important strategy for diabetes control and maintenance in the optimal range includes patient compliance with self-care, such as blood glucose monitoring, foot care performance, and healthy lifestyle adherence [4].

Self-care adherence behaviors are reported to be poor in diabetic patients with physical disabilities [5]. Considering that the mortality rates caused by diabetes were 7.7 times higher in the population with disability [6], diabetes has accounted for a large part of premature death in the population with disabilities. Self-care adherence is used to describe the congruence between the recommended practices by healthcare providers and actual behaviors [7]. The ability to engage in self-care therefore can be compromised when a patient is unable to fully understand their diagnosis and treatment [8]. Thus, the patient’s ability to find, understand, and act on health-related information, also known as health literacy, is a determinant of the patient’s self-care behavior [9].

People with physical disabilities are the minority group that is vulnerable to inequality in health, thus understanding disability and health is critically important. Populations with a disability still have largely been excluded from the research sector due to low priority and less attention despite the calls for emerging disability studies related to their health issues to prevent health disparity [10]. Considering that the disabled population is growing worldwide due to medical advances and the aging process, Rio et al. [10] asserted that disability should be treated as a demographic factor, such as sex, age, or ethnicity, in research. Therefore, the present study presented a novel approach to addressing their health literacy, which is well known as a health determinant, and diabetes self-care behavior concerning multiple factors in people with physical disabilities who have diabetes.

Health literacy is defined as having “the cognitive and social skills, which determine the motivation and ability of individuals to gain access to, understand, and use information in ways that promote and maintain good health” [11]. Evidence has indicated that health literacy can affect one’s ability to understand health issues [8], communicate with healthcare providers [12], make health-related decisions, and use health services [13]. Additionally, health literacy has been theorized as one important, nonclinical factor that is associated with diabetes self-care behavior. The growing body of research has indicated that health literacy is a determinant of glycemic control [14], medication adherence [15], and diabetic control lifestyle adherence, such as blood glucose monitoring, foot care, and diet [16].

Health literacy empowers people with skills to improve their health and has important medical and societal implications [17]. Population with physical disabilities may have the greatest need for health literacy as they often encounter diverse and complex barriers to healthcare services and have special self-care regimens to follow [18, 19]. Physical impairments are one of the risk factors for low health literacy [20]; thus, health literacy issue is more common within these vulnerable population [18]. Nonetheless, the vast majority of the health literacy studies have been focused on the general population, and the population with physical disabilities has been underserved in the corresponding research area.

Paasche-Orlow and Wolf proposed a causal pathways conceptual model explaining that the linkage of health literacy to health outcomes could be mediated by a range of health actions at systematic, interactional, and self-care levels [21]. Each level of mechanisms is illustrated with three distinctive points, including access and use of healthcare, provider-patient interactions, and patient self-care, and these points, in turn, influence each other. Each respective point involves different patient factors. Patient factors, such as a patient’s navigational skills, self-efficacy, and perceived barriers to care, can be affected by health literacy at the point of access and use of healthcare. Factors involve knowledge, beliefs, and participation in decision-making in provider-patient interactions. Patient factors comprise motivation, self-efficacy, problem-solving, and knowledge and skills, which can affect self-care performance, at the point of self-care.

This model is unique because it highlights that the link between health literacy and its outcome is due to not only individual attributes at the self-care level, such as self-efficacy, and motivation, but also systematic and interactional aspect attributes, including access and use of healthcare and provider-patient interactions [21]. Substantial empirical attention has been established on the effect of health literacy on self-efficacy, motivation, or knowledge at self-care levels [22–25]. However, almost no research has extensively included the systematic and interactional aspects of this model. Therefore, validating the applicability of Paasche-Orlow and Wolf’s pathway model in predicting the linkage between health literacy and self-care that goes beyond previous research by including four mediators, i.e., access to healthcare, provider-patient interactions, motivation, and self-efficacy, is novel in diabetic adults with physical disabilities.

The relationship between health literacy, self-care behaviors, and related mediators may also be necessary to explore the moderating variable. Age may be a potential moderator of these relationships by considering the different sensitivities to health literacy and self-care in patients with different characteristics. Previous studies have indicated that advanced age was independently associated with lower compliance to treatment regimens [26]. Another study revealed that younger patients were most likely knowledgeable in self-care diabetes, leading to diabetes management adherence [27]. Similarly, the impact of health literacy on the health outcome of older people is significantly higher than on those of younger counterparts [28]. Therefore, a hypothesis that age would moderate the direct and indirect effects in the hypothetical model was added.

**Fig 1:**
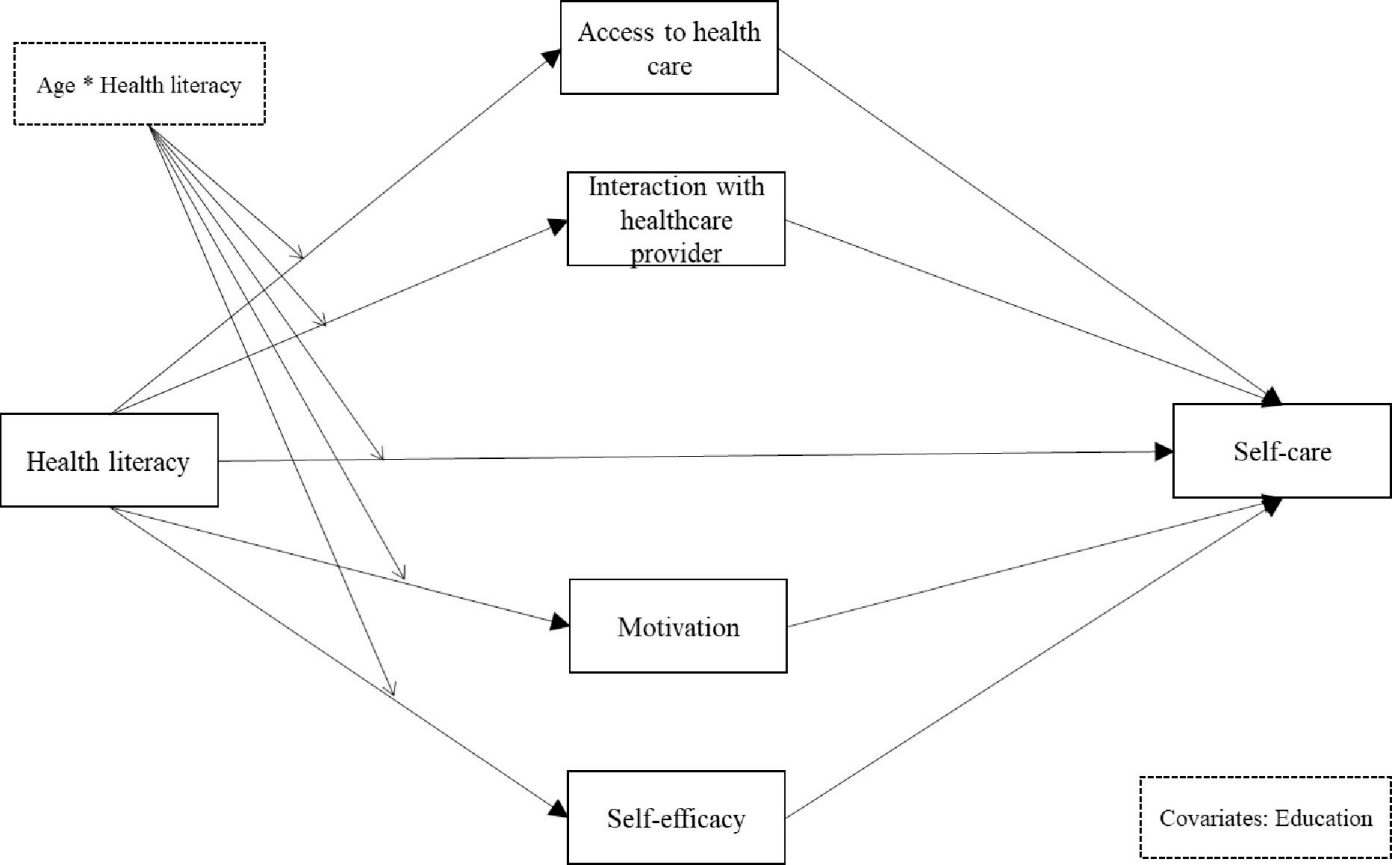
Moderated medication model of health literacy on self-care through multiple mediators moderated by age.

The present study aims (a) to explore the direct and indirect effects of health literacy on diabetes self-care through healthcare access, provider-patient interaction, diabetes control motivation, and diabetes control self-efficacy based on Paasche-Orlow and Wolf’s pathway model and (b) to investigate the moderating effect of age in this moderated mediation model. Fig 1 illustrates the conceptual model.

## Methods

### Patient and public involvement

Patients or the public were not involved in the design, conduct, reporting or dissemination of the research.

### Design

This study used the cross-sectional exploratory design.

### Sample/Participants

The inclusion criteria were as follows: (a) 18 years of age or older; (b) a person who was diagnosed with a physical disability and possessed a Handicapped Welfare Card or certificate of disability registration that is provided by the Ministry of Health and Welfare; (c) a person who was diagnosed with type 2 diabetes; and (d) absence of blindness and intellectual disability. Physical disability is defined as a permanent physical impairment resulting from amputation, joint disorder, limb deformity, spinal cord injury, or a motor disturbance according to the criteria of the Korean disability registration system [29, 30].

The recruitment advertisements were placed on an online community website, where 8,912 members share a common interest in physical disability and most of them have physical disabilities. Among them, the participants who met the study criteria autonomously participated in our study and were asked to self-declare whether they met the eligibility criteria. In total, 230 patients participated in this study, and 16 patients were excluded due to duplicate participation. Ultimately, 214 participants were included in the analysis. Fritz and Mackinnon’s mediation model simulations were applied to determine the sample size, with medium effect sizes (d = 0.26) for mediation paths and a power of 0.80 in a mediation model, which yield a sample size of 148 [31]. Additionally, the sample size should be >10 times the observed variables [32]; thus, a sample size of 214 met the requirement for testing the hypothesized models.

Convenience sampling was employed to recruit diabetic patients with physical disabilities for this study. Despite its advantages in accessibility and efficiency, this sampling method is susceptible to sample biases, systematic errors, limited representativeness, and reduced generalizability of research findings [33]. It is imperative to acknowledge the inherent limitations associated with convenience sampling, which may impact the extrapolation of findings beyond the sampled population.

### Data collection

A cross-sectional online survey was conducted from August 23 to September 17, 2021. Data were collected using a structured questionnaire about the hypothetical model components of this study. This study reports data from a survey on health literacy, diabetes self-care, healthcare access, provider-patient interaction, diabetes control motivation, and diabetes control self-efficacy in diabetic patients with physical disabilities.

### Ethical considerations

The study was approved by the Institutional Review Board at a university (IRB No. 2108/003-019). The study was conducted following the Declaration of Helsinki and in compliance with IRB regulations. Among the participants who accessed the online survey link, only those who had read and agreed to provide written informed consent participated in the survey.

### Data analysis

Descriptive statistics, including frequencies, percentages, mean, and standard deviations (SD), were used to describe sample characteristics and summarize study variables. The Pearson correlation analysis was performed to identify the association among the main research variables. Hayes’s PROCESS macro for SPSS was used to test the hypothesized moderated mediation model (Fig 1). PROCESS probes mediation and moderation effects using bias-corrected bootstrapping in a regression framework. PROCESS also calculates interactions to determine the conditional direct or indirect effects on different moderator levels. PROCESS provides a specific index of moderated mediation. Statistical significance of this value means the presence of moderated mediation effect. First, model 4 of PROCESS was used to test the mediating roles of 4 mediators in the relationship between health literacy and diabetes self-care by generating a bias-corrected bootstrap confidence interval using 5,000 bootstrapping samples. Second, the moderated mediation model was tested with model 8 by age groups (young adults aged <40 and midlife adults aged ≥40) as moderators [34]. Education was treated as a controlled variable in the hypothetical model considering the influence of education on health literacy and self-care behavior [16]. The threshold of value of α was adjusted to less than 0.025, deviating from the conventional 0.05, aiming to mitigate the risk of Type I errors [35].

### Validity and reliability/rigor

#### Health literacy

Health literacy was assessed using the revised Korean Health Literacy Assessment Tool (KHLAT-4); which is the Korean modified version of the Rapid Estimate of Adult Literacy in Medicine (REALM) [36]. KHLAT is a word recognition test of common medical words and layman terms relating to physiology and illnesses [37] and was developed by incorporating culturally appropriate translations for the 66 words from the REALM and modifying its administration and rating [38]. Participants responded to a written questionnaire that asked whether they knew each of the 66 words using a 4-point Likert scale (1 = I don’t know this term, 2 = I have seen the term before but don’t know the meaning, 3 = I have seen the term before and know its meaning a little, 4 = I know this term) instead of reading each of the words aloud to score the number of correctly pronounced words. The response were reclassified into either 1, which equated to knowing the word (including only 4 = I know this term) or 0 which equated to not knowing the word. The total score range was 0–66, and higher scores indicated a higher level of health literacy. The internal consistency of the measurement for this sample was ɑ = 0.95

#### Diabetes self-care

Diabetes self-care behaviors were assessed using the validated 15-item Diabetes Self-care Scale [39]. The scale asks the participants to indicate how often they perform certain self-care behaviors regarding diabetic diet, weight control, medication adherence, and physical activity. Each item was scored on a 4-point Likert scale. The total score ranges from 15 to 60, with higher scores indicating higher compliance in terms of diabetes self-care behavior. The Cronbach’s alpha of the tool was 0.83 in Gu’s study [39] and 0.80 in this study.

#### Access to healthcare

Healthcare access served as our measure of access, and healthcare utilization was assessed using the Health Navigation Self-sufficiency Scale [40]. The tool contains 10 items that intend to quantify the degree of self-sufficiency of individuals when navigating healthcare systems, cover health management, health information, seeking, finding a doctor and making an appointment, transportation, communication with healthcare providers, mediation use, medical expense payment, reading and filling out medical documents, and medical decision-making. Participants were asked to indicate whether they did a specific task on their own or if they needed someone to assist them with each item. A response that indicate self-sufficiency in performing the task was coded as 1 and a need for assistance as 0. The scores range from 0 to 10, with higher scores indicating greater healthcare navigation self-sufficiency. The reliability coefficient of the scale was 0.73 in the original research [40] and 0.75 in this study.

#### Provider-patient interactions

Provider-patient interactions refer to a tendency to be actively involved in shared decision-making and communication with healthcare providers. Provider-patient interactions were assessed using a Medical Decision-making Tool [41]. The tool contains seven items that inquire about shared decision-making, such as doctor-patient communication, doctor’s expertise, shared health information, and the understanding of treatments or medical tests. Each item was evaluated on a 5-point Likert scale, where the scores range from 5 to 35, with higher scores indicating a higher degree of medical decision-making involvement. The internal consistency score of the scale was ɑ = 0.83 in Kim’s study [41] and 0.78 in this study.

#### Diabetes control motivation

This represents attitudes about the outcomes of performing diabetes self-care behaviors and was measured by the Diabetes Self-management Attitude Scale [42]. This scale contains 10 items that are rated on a 5-point Likert scale. Scores for each item range from 1 for “strongly disagree” to 5 for “strongly agree,” and the scores range from 10 to 50, with higher scores indicating a more positive attitude about the self-care behavior outcomes. The reliability coefficient of this scale was 0.95 as the scale was developed [42] and 0.82 in this sample.

#### Diabetes control self-efficacy

Diabetes control self-efficacy was measured by the Korean modified version of the Self-Efficacy for Diabetes Scale [43], which was developed by incorporating culturally appropriate translations by Kang [44]. The scale contains 8 items that ask individuals about their confidence in doing specific activities related to diabetes, such as diet, exercise, blood sugar, and diabetes management. Scores for each item correspond to one’s confidence in performing the activities regularly, ranging from 1 “not at all confident” to 10 “totally confident,” where the scores range from 8 to 80. The descriptors only anchor the beginning point (1) and end of the scale (10). Reported internal consistency reliability coefficients were 0.89 in Kang’s study [44] and 0.68 in this study.

#### Demographic and health-related characteristics

Demographic characteristics, such as age, gender, education, and monthly household income, and health-related characteristics, including disability severity, functional limitation, diabetes duration, and treatment regime, were collected. Disability severity was assessed by asking about their disability degree according to the Korean disability registration system. Functional limitation was measured using the Washington Group Short-set on Functioning (WG-SS) [45]. WG-SS consists of six items that ask about difficulties that the respondents may have in doing specific activities, such as seeing, hearing, walking, remembering or concentrating, washing or dressing, and communicating due to a health problem.

## Results/Findings

### Participant characteristics

Of 214 survey results, none had missing data or numerical outliers. Participant characteristics are presented in Table 1. Of the participants, 43.5% were aged <40 years and 56.5% were 40 years or over. The mean age of the participants was 41.4 (SD 7.41) years, approximately 24–66 years. Approximately half (52.3%) were males, most had an educational level of high school (42.5%) or above (54.2%), and 41.1% had less than 2,000,000 Korean won. The majority of them (84.1%) had a severe physical disability and 63.1% reported difficulty in at least one of the six core domains in WG-SS. All participants were diagnosed with type 2 diabetes, with a mean duration of 50.16 (SD 34.91) months. Of the participants, 79% were taking oral hypoglycemia agents. The mean score for health literacy, diabetes self-care, and healthcare access was 41.04 (SD 19.48), 38.99 (SD 7.52), and 6.77 (SD 2.52), respectively. The mean scores of provider-patient interactions, diabetes control motivation, and diabetes control self-efficacy were 21.75 (SD 6.13), 32.49 (SD 8.21), and 46.14 (SD 12.97), respectively.

**Table 1:** Characteristics of participants *(N* = 214).

	Variables	*n* (%) or Mean±SD	Range (Min.~Max.)
Gender	Male	112 (52.3)	
	Female	102 (47.7)	
Age (years)		41.4 ±7.41	24~66
	<40	93 (43.5)	34.16±2.93
	≥40	121 (56.5)	46.96±4.37
Education	≤Middle school	7 (3.3)	
	High school	91 (42.5)	
	≥College	116 (54.2)	
Monthly household income (KRW10,000)	<200	88 (41.1)	
	200~299	25 (11.7)	
	300~399	20 (9.3)	
	400~499	31 (14.5)	
	≥500	50 (23.4)	
Disability severity^a^	Mild	180 (84.1)	
	Severe	34 (15.9)	
Functional limitation^b^	No	79 (36.9)	
	Yes	135 (63.1)	
Duration of Diabetes (month)		50.16±34.91	
Treatment regime^c^	Lifestyle management	55 (25.7)	
	Oral medication	169 (79.0)	
	Insulin injection	72 (33.6)	
Health literacy		41.04±19.48	8~66
Diabetes self-care		38.99±7.52	18~60
Access to healthcare		6.77±2.52	1~10
Provider-patient interaction		21.75±6.13	7~35
Diabetes control motivation		32.49±8.21	11~50
Diabetes control self-efficacy		46.14±12.97	8~80

^a^According to the Korean disability registration system

^b^Respondents reporting “some difficulty”, “a lot of difficulty”, or “cannot do at all” in at least one of the six core domains in the Washington Group Short Set on Functioning

^c^Responding duplicate answers allowed

### Correlation analysis of research variables

Table 2 displays bivariate correlations for all variables of interest. Of note, the variables showed significantly moderate positive correlations with each other, and all falling below the threshold of 0.7, without indicating multicollinearity.

**Table 2:** Correlations among the main variables.

	Health literacy	Access to healthcare	Provider-patient interaction	Diabetes control motivation	Diabetes control self-efficacy
	*r*				
Access to healthcare	.63***				
Provider-patient interaction	.52***	.40***			
Diabetes control attitude	.51***	.24***	.65***		
Diabetes control self-efficacy	.44***	.22***	.50***	.51***	
Diabetes self-care	.50***	.26***	.49**	.53***	.63***

***p < .001

### Parallel multiple-mediation model analysis

The direct effect of health literacy on diabetes self-care and the indirect effects through healthcare access, provider-patient interaction, diabetes control motivation, and diabetes control self-efficacy when controlling for education are summarized in Table 3. Results from bootstrapping yielded a significant indirect effect of health literacy on diabetes self-care through diabetes control motivation (*β* = 0.033, 95% Confidence interval [CI] 0.006~0.065) and diabetes control self-efficacy (*β* = 0.070, CI 0.035~0.095). Meanwhile, healthcare access and provider-patient interaction exhibited no significant mediating effects between health literacy and diabetes self-care. Thus, the results confirmed that diabetes control motivation and diabetes control self-efficacy partially mediated the relationship between health literacy and diabetes self-care, because of the positive direct effect of health literacy on diabetes self-care (*β* = 0.081, CI 0.054~0.169). The indirect to the direct effect ratio was 0.57, indicating that the indirect effect was 57% of the total effect size.

**Table 3:** Mediating effects between health literacy and self-care with education as covariate (*N* = 214).

Path		Effect	SE	t	*p*	R^2^	F(p)
Step I	Health literacy → Diabetes self-care	0.081	0.030	2.73	.007	0.49	33.014 (< .001)
Step II	Health literacy → Access to Healthcare	0.059	0.007	7.58	< .001	0.46	91.08 (< .001)
	Health literacy → Provider-patient interaction	0.169	0.022	7.62	< .001	0.27	39.00 (< .001)
	Health literacy → Diabetes control motivation	0.210	0.030	7.02	< .001	0.26	36.35 (< .001)
	Health literacy → Diabetes control self-efficacy	0.289	0.049	5.89	< .001	0.19	25.45 (< .001)
Step III	Health literacy → Diabetes self-care	0.189	0.027	6.92	< .001	0.25	35.70 (< .001)
	Access to Healthcare → Diabetes self-care	-0.139	0.211	-0.66	.510		
	Provider-patient interaction → Diabetes self-care	0.086	0.088	0.97	.334		
	Diabetes control motivation → Diabetes self-care	0.156	0.065	2.38	.018		
	Diabetes control self-efficacy → Diabetes self-care	0.382	0.851	0.45	.654		
Path					Effect	SE	95% CI^a^
Indirect effect	Health literacy → Access to Healthcare → Diabetes self-care			−0.008	0.0127	−0.033~0.018	
	Health literacy → Provider-patient interaction → Diabetes self-care			0.014	0.0185	−0.024~0.049	
	Health literacy → Diabetes control motivation → Diabetes self-care			0.033	0.0153	0.006~0.065	
	Health literacy → Diabetes control self-efficacy → Diabetes self-care			0.070	0.0192	0.035~0.095	
Total Indirect effect				0.109	0.029	0.054~0.169	
Direct effect of Health literacy on Diabetes self-care				0.081	0.030	0.023~0.139	
Total effect of Health literacy on Diabetes self-care				0.190	0.027	0.135~0.244	

^a^The significance of the indirect effects was calculated with bias-corrected intervals (0.95) bootstrap analysis with a bootstrap sample of 5000

### Conditional process model analysis

PROCESS macro (model 8) was adopted to further examine the direct and indirect effects of health literacy on diabetes self-care when moderated by age groups (i.e., ages of <40 and ≥40). Table 4 shows the interactive action between health literacy and age, which was not associated with the direct effect of health literacy on self-care, after controlling for education (*β* = 0.015, *p* = .778). Significant interactive actions were associated with the mediators, including provider-patient interaction (*β* = −0.017, *p* = .001), motivation (*β* = −0.364, *p <* .001), and self-efficacy (*β* = −0.267, *p* = .007). Specifically, age alone did not affect motivation (*β* = 1.524, *p* = .172) or self-efficacy (*β* = 2.032, *p* = .305), but the interaction between health literacy and age was significant, confirming the moderating effect of age. The conditional indirect effects were significant for motivation and self-efficacy when moderated by age. The overall size (moderated mediation index) of the conditional indirect effect was −0.0588 (CI −0.117~−0.012) for motivation and −0.0646 (CI −0.127~−0.011) for self-efficacy. Age functioned as a mediating effect moderator of diabetes control motivation and diabetes control self-efficacy between health literacy and diabetes self-care.

**Table 4:** Regression coefficient and conditional effect of age (young adults <40 and midlife adults ≥40) with education as covariate.

	Dependent variable	Independent variable	Effect		SE	*t*	*p*	R^2^	F(*p*)
Step I	Access to healthcare	Health literacy	0.053		0.008	6.26	< .001	0.48	47.78 (< .001)
		Age	0.623		0.315	1.97	.050		
		Health literacy * Age	−0.020		0.016	−1.28	.203		
	Provider-patient interaction	Health literacy	0.144		0.023	6.30	< .001	0.43	27.18 (< .001)
		Age	2.220		0.862	2.58	.011		
		Health literacy * Age	−0.017		0.043	−3.94	.001		
	Diabetes control motivation	Health literacy	0.191		0.030	6.47	< .001	0.39	33.33 (< .001)
		Age	1.524		1.111	1.37	.172		
		Health literacy * Age	−0.364		0.056	−6.56	< .001		
	Diabetes control self-efficacy	Health literacy	0.266		0.053	5.07	< .001	0.23	15.27 (< .001)
		Age	2.032		1.977	1.03	.305		
		Health literacy * Age	−0.267		0.099	−2.70	.007		
Step II	Diabetes self-care	Access to healthcare	−0.142		0.216	−0.66	.511	0.49	24.60 (< .001)
		Interaction with healthcare providers	0.081		0.089	0.91	.364		
		Diabetes control motivation	0.162		0.070	2.31	.022		
		Diabetes control self-efficacy	0.242		0.036	6.80	< .001		
		Health literacy	0.076		0.031	2.44	.016		
		Age	0.456		0.962	0.47	.636		
		Health literacy * Age	0.015		0.052	0.28	.778		
Conditional Direct effect		Age	Direct effect			Boot SE		BootLLCI	BootULCI
Self-care		Young adults (age<40)	.0677			.0472		−0.025	0.161
		Midlife adults (age≥40)	.0825			.0344		0.147	0.150
Conditional indirect effect		Age	Indirect effect			Boot SE		BootLLCI	BootULCI
Access to healthcare		Young adults (age<40)	−0.009			0.014		−0.036	0.019
		Midlife adults (age≥40)	−0.006			0.010		−0.026	0.013
		Moderated mediation index		.0029				−0.008	0.015
Provider-patient interaction		Young adults (age<40)	0.019			0.027		−0.036	0.072
		Midlife adults (age≥40)	0.006			0.008		−0.011	0.023
		Moderated mediation index		−.0138				−0.055	0.025
Diabetes control motivation		Young adults (age<40)	0.064			0.029		0.013	0.125
		Midlife adults (age≥40)	0.005			0.006		−0.006	0.019
		Moderated mediation index		−.0588				−0.117	−0.012
Diabetes control self-efficacy		Young adults (age<40)	0.101			0.030		0.047	0.164
		Midlife adults (age≥40)	0.036			0.018		0.003	0.072
		Moderated mediation index		−.0646				−0.129	−0.011

SE = standard error; LLCI = the lower limit confidence interval of effects; ULCI = the upper limit confidence interval of effects

**Fig 2:**
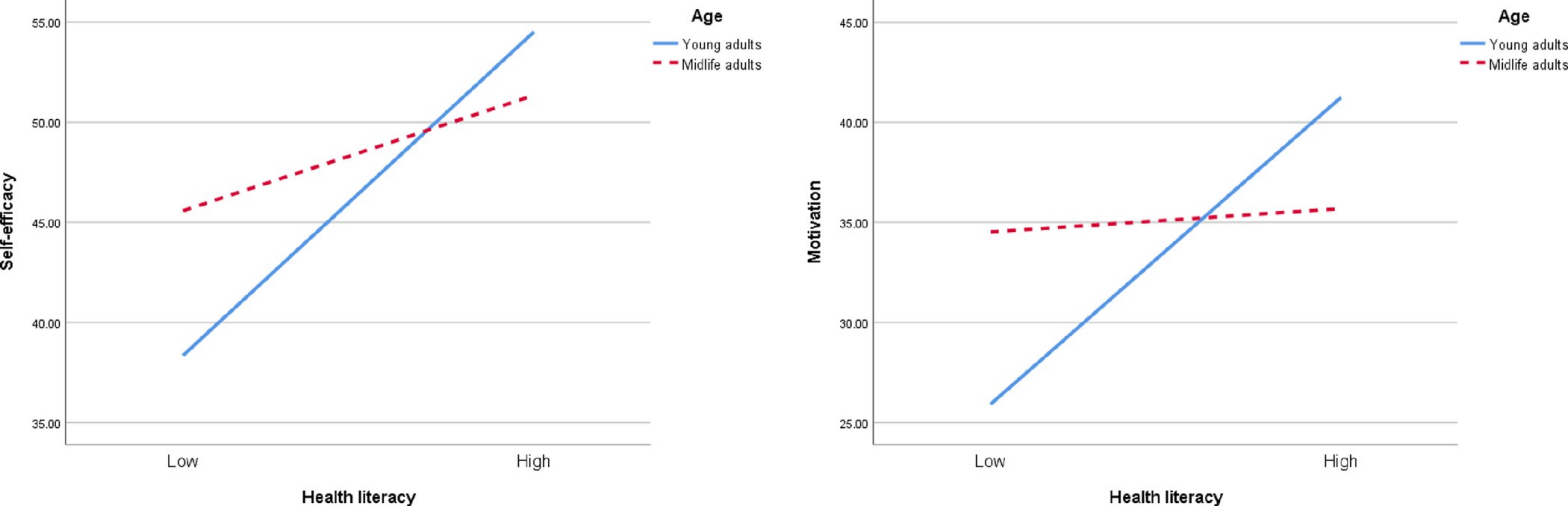
The interactions between health literacy and age on motivation, self-efficacy, and self-care with education as covariates.

A test of simple slopes was conducted to demonstrate the significant interaction at ages of <40 and ≥40. Young adults (age <40) showed a positive association between health literacy and motivation (*B*_simple_ = 0.064, CI 0.013~0.125); on the other hand, there was no association in midlife adults (age of ≥40; *B*_simple_ = 0.005, CI −0.006~0.019). Meanwhile, higher health literacy was associated with higher diabetes self-efficacy in young adults (*B*_simple_ = 0.101, CI 0.047~0.164). The effect of health literacy and self-efficacy remained significant in midlife adults although considerably weaker (*B*_simple_ = 0.036, CI 0.003~0.072). Fig 2 demonstrated age as a moderator of the relationships between motivation, self-efficacy, and health literacy.

## Discussion

The present study has explored the potential pathways that link health literacy to diabetes self-care behavior via theoretically selected mediators, i.e., healthcare access, provider-patient interactions, diabetes control motivation, and diabetes control self-efficacy, which were moderated by age, considering the growing calls for theory-driven research on health literacy. the impact of health literacy on diabetes self-care behaviors among diabetic patients without disabilities has been confirmed in many studies [16, 46]; however, research on people with physical disabilities is limited. Notably, this study may be the first theory-based research to uncover the potential mediators and the moderator of the relationship between health literacy and self-care behavior in type 2 diabetic patients with physical disabilities. Our moderated mediation model has partially supported the hypotheses and contributed to a better understanding of the association between health literacy and self-care, which helps improve health behaviors and ultimately decreases the burden of diabetes in such vulnerable populations.

The participants in this study appeared to have an average diabetes self-care score of 38.99. This was relatively low, compared to those of older adults with diabetes in other studies, whose mean scores ranged between 45.45 and 50.26 [47, 48]. The low diabetes self-care rate in this sample has emphasized that people with physical disabilities are likely at higher risk for complications due to uncontrolled diabetes. The vast majority of day-to-day diabetes self-care activities are undertaken by the patients on their own [4]; hence, developing strategies that include physically disabled people is urgently necessary to fully participate in self-care activities. Health literacy can play a key role in understanding barriers to participation and facilitators for building partnerships that support people with disabilities [19]. Therefore, this study would contribute to a comprehensive understanding of self-care behavior determinants, including health literacy and relating factors at the individual, interactive, and systematic levels.

Our study showed that health literacy was directly related to diabetes self-care activities in the mediation model analysis. Some studies have found that health literacy predicted diabetes self-care behaviors [16, 46, 49], whereas others revealed no association [50, 51]. A meta-analysis argued varying relationships between health literacy and diabetes self-care based on the types of health literacy tools [52]. For example, the association with self-care activities was significant in studies that used perception-based health literacy measures, whereas studies with performance-based tests revealed no such association. However, this remained conflicting with our study results, which proposed the significant positive associations with self-care activities using performance-based health literacy measures. The inconsistent results may be attributed to the different population characteristics since most of the studies included in Marciano et al’ [52]s meta-analysis were conducted on the population without disabilities. The evidence for the association between health literacy and diabetes self-care is very limited and may vary within the types of measure [53]; thus, further studies are needed to produce more evidence, especially in people with disabilities.

The proposed mediator analysis indicated a significant mediating effect in diabetes control motivation, allowing health literacy to be indirectly related to diabetes self-care. This result was consistent with prior studies that show the association between motivation and health literacy and diabetes-controlling activities [54, 55]. Hussain [56] also asserted the congruent result through an intervention study that aimed to improve health literacy to enhance motivation and commitment toward diabetes self-care activities. Nutbeam [57] emphasized the relationship with motivation in ways that promote health by extending the definition of health literacy as an action-orientated concept rather than simply an intellectual capacity. Health literacy is directed toward improving personal capacity to independently act on knowledge for motivation improvement and acting on received advice [58]. Therefore, improving health literacy can, in turn, promote motivation and change the health behavior of people with diabetes. Thus, health literacy may be an important driver for improving motivation, which results in recommended self-care behavior adherence, in patients with diabetes with physical disabilities.

Similarly, our result has indicated that self-efficacy for diabetes control plays a mediating role in the relationship between health literacy and diabetes self-care. The association between health literacy and self-efficacy, which was positive predictors of good diabetes control behaviors, was congruent with previous research findings that highlight self-efficacy as a factor that was positively associated with health literacy and self-care activities, such as dietary, foot care, and exercise [24, 38]. The way people behave can often be better predicted by the beliefs that they hold about their capabilities than by what they are capable of accomplishing because self-efficacy perceptions help determine what individuals do with the knowledge and skills that they have [59]. Thus, both health literacy and self-efficacy are vital constructs in diabetes care. Health literacy is conceptualized as having an impact on knowledge and skill attainment levels, with greater knowledge and skill levels impacting the self-efficacy level and diabetes self-care activities observed by individuals [24]. Self-efficacy alone is unable to generate good diabetes self-care behavior without behavioral capabilities, including knowledge or skills in performing a specific task, such as blood glucose management [60]. This result implies that patients who have a good understanding and extract health information are more likely to have confidence in their ability to perform diabetes-related health behaviors while those with low health literacy may feel less confident.

Contrarily, no significant mediating effects were found in healthcare access and provider-patient interactions. The reasons for the insignificant associations shown in this study are uncertain, but likely associations can be deduced. First, low health literacy is related to the deprived ability to choose or navigate appropriate healthcare professionals at the right time, thereby mainly hindering access in the general population [13]. Meanwhile, additional issues and challenges confronted people with disabilities in accessing healthcare services, which are different compared to people without disabilities [61]. The physical or geographical barriers are the foremost access barriers in the physically disabled population due to their physical limitations, regardless of their health literacy level [62]. These barriers include lack of accessible equipment, lack of hospital transportation, and physical barriers to entering and using healthcare facilities [62]. The issues of healthcare-related access to health literacy in people with physical disabilities should be viewed from a more comprehensive perspective considering various barriers that they confront.

Furthermore, provider-patient interactions are a process in which the provider and patient exchange information, build relationships, and participate in shared decision-making about medical care, which is often influenced by the patient’s health literacy level [12]. Not only the patient’s health literacy level but also the attitude of a provider may influence the quality of interactions because the interactions go both ways. One of the key determinants of provider-patient interactions for people with disabilities is provider perspectives and understanding of the disability [63]. Providers and staff may have negative attitudes toward disability, such as the view that a person’s disability is a negative trait and a tendency to attribute everything to the disability, and ignore new complaints [64]. It can cause misunderstandings and discordant expectations between providers and disabled patients and negative impact on the provider-patient interactions. Examining the impact of such issues on the provider-patient interaction concerning health literacy status among people with disabilities is worthwhile in future studies because attitudes of providers toward people with disabilities remained a significant deterrent to good-quality interactions.

Another important study finding was that age moderated the mediating role of diabetes control motivation and diabetes control self-efficacy in health literacy and self-care association. Specifically, the health literacy of young adults positively predicted motivation, whereas no significant effect in midlife adults. Additionally, health literacy in young adults had a stronger impact on self-efficacy than in midlife adults. Those belonging to the younger age group in this study are likely newly diagnosed with diabetes or have a shorter diabetes duration because the age of onset for type 2 diabetes is an average of 45 year-old [3]. Therefore, people who are new to diabetes need more information and understanding to accept the diagnosis and enhance diabetes management motivation and self-efficacy [65]. Thus, health literacy may have played a more significant role in young adults. Furthermore, self-care behaviors may already have become habitual in midlife adults, thereby resulting weaker health literacy impact on them because lifestyle behavior is often guided by habits [66]. The moderating mechanisms of age are inferred only, but it suggests that efforts to improve health literacy among young adults with physical disabilities may be the most effective means to reduce health disparities and improve outcomes. Healthcare providers should make efforts to provide interventions to promote health literacy-related self-care as early as possible, and patient demographic characteristics should be considered to potentially derive benefit from such interventions.

### Limitations

Several limitations must be acknowledged in this study. First, the cross-sectional nature of this study could limit causal inferences although the path analysis hypothesized causal relationships between the interested variables based on the theory. Therefore, additional longitudinal and experimental studies are required to confirm the causal pathways in the final model. Second, the participants were recruited using convenience sampling from an online website to reduce direct contact with the participants during the coronavirus disease-2019 pandemic. The participants were required to use the Internet, which might exclude people who have a limited ability to use the Internet. The participants in this study showed higher health literacy than those in previous studies because the ability to use the Internet is highly related to high-health literacy [67–69]. Thus generalizing the study results must be made with caution, and future studies should expand the accessible population and sampling methods to increase generalizability. Moreover, despite its practicality, convenience sampling is prone to biases, potential systematic errors, limited representativeness, and reduced generalizability of research findings [33]. To mitigate these limitations, further investigations are warranted. Future studies should endeavor to minize bias and uncertainty inherent in convenience sampling by employing larger datasets and validating research outcomes. Additionally, researchers should add diversity to mend convenience samples by achieve an appropriate cross-section of the target population. Thirdly, while the threshold of value of α was adjusted to less than 0.025 to mitigate the risk of Type I errors in this study, employing even more stringent correction methods like Bonferroni or Benjamini-Hochberg adjustments is recommended to comrephensively address multiple comparison issues [70]. Forth, our results uncovered few significant findings; however, the effect sizes were small. Therefore, repetitive studies are required to confirm the significant findings because small effect sizes indicate limited practical application. Lastly, KHLAT-4 is a widely used health literacy measure that assesses the understanding of medical terms; however, it does not capture other skills that may include numeracy, oral communication, and memory. Hence, further research is required to confirm the findings using different health literacy tools.

## Conclusions

This study adds theory-based, empirically tested evidence to the literature on diabetes self-care in people with physical disabilities by depicting the influence of health literacy on diabetes self-care behavior through the factors at the systematic (access to healthcare), interactive (provider-patient interaction), and individual levels (diabetes control motivation and self-efficacy), moderated by age. Our findings indicated the mediating roles of motivation and self-efficacy in linking health literacy with self-care behaviors, as well as the direct effect of health literacy on self-care behaviors. Our moderated mediation model can provide useful knowledge for healthcare providers to design theory-based intervention programs for diabetic patients with physical disabilities. Tailoring the educational intervention to the individual level of health literacy may be an effective strategy to improve motivation and self-efficacy to perform diabetes self-care activities among people with physical disabilities. Furthermore, age can moderate the mediating role of motivation and self-efficacy between health literacy and self-care, especially for young adults. Therefore, health literacy evaluation and improvement in young-adult patients with physical disabilities should not be ignored in global health management.

## Supporting information

S1 Data (XLS)
